# Climate change and influenza: the likelihood of early and severe influenza seasons following warmer than average winters

**DOI:** 10.1371/currents.flu.3679b56a3a5313dc7c043fb944c6f138

**Published:** 2013-01-28

**Authors:** Sherry Towers, Gerardo Chowell, Rasheed Hameed, Matthew Jastrebski, Maryam Khan, Jonathan Meeks, Anuj Mubayi, George Harris

**Affiliations:** Arizona State UniversityArizona State University; Arizona State University, Tempe, Arizona; Fogarty International Center, National Institues of Health, Bethesda, MD, USA; Northeastern Illinois UniversityNortheastern Illinois University; Northeastern Illinois UniversityNortheastern Illinois University; Northeastern Illinois UniversityNortheastern Illinois University; Northeastern Illinois University; Northeastern Illinois University; Northeastern Illinois UniversityNortheastern Illinois University

## Abstract

The 2012-13 influenza season had an unusually early and severe start in the US, succeeding the record mild 2011-12 influenza season, which occurred during the fourth warmest winter on record. Our analysis of climate and past US influenza epidemic seasons between 1997-98 to present indicates that warm winters tend to be followed by severe epidemics with early onset, and that these patterns are seen for both influenza A and B. We posit that fewer people are infected with influenza during warm winters, thereby leaving an unnaturally large fraction of susceptible individuals in the population going into the next season, which can lead to early and severe epidemics. In the event of continued global warming, warm winters such as that of 2011-12 are expected to occur more frequently. Our results thus suggest that expedited manufacture and distribution of influenza vaccines after mild winters has the potential to mitigate the severity of future influenza epidemics.

## Introduction

The 2011-12 influenza season was unusually late and mild, and set a new record for the season with the lowest and latest peak of influenza-like illness (ILI) since surveillance efforts began 1. While the underlying causative dynamics of the severity and timing of influenza epidemics are multi-faceted, a primary contributing factor to the mildness of the 2011-12 season was likely the fact that the national meteorological winter of 2011-12 was the fourth warmest on record 2; several prior studies have shown that influenza transmissibility sharply decreases in warmer temperatures and/or high humidity (e.g., 3 4 5 6 7 8).

In contrast to the 2011-12 season, the ongoing 2012-13 season is off to an unusually early and severe start, despite the fact that the national climate this past autumn was close to the seasonal average. Here we analyzed the weekly time series of confirmed influenza cases in the US from the 1997-98 influenza season to present. Our findings indicate that influenza epidemic severity and time of onset is significantly associated with the average winter temperature during the previous season, with severe and early influenza seasons being much more likely following a mild winter.

In the event of continued global warming, warmer than average winters are expected to occur more frequently, but variability in seasonal temperatures will of course remain, and average winters will still occur with regularity for some time to come. Our work suggests that mild influenza seasons during unusually warm winters are a harbinger of the likelihood of an unusually severe season to come. Hence, these findings could guide improved prevention efforts, including progressive vaccination programs after a mild winter to achieve high vaccination coverage well in advance of the next influenza season.

## Methods and Materials

**Sources of data:** From the CDC website we obtained the weekly time series of laboratory-confirmed influenza incidence recorded across ten US geographic regions between the 1997-98 to 2012-13 influenza seasons (the time period over which data was available) 9. In addition, we obtained the antigenic characterization of the dominant strains of each influenza season and the percentage of isolates that matched the components of the influenza vaccine for that season 9. We also obtained the overall influenza-related mortality during each season using data obtained from the CDC 122 Cities Mortality Reporting system 10.

Time series of influenza B, A(H1N1) and A(H3N2) for annual seasons between 1997-98 to 2012-13 are shown in Figure 1. Both influenza B and A(H3N2) epidemics are off to an extraordinarily early start this season, made even more remarkable for the fact that the vaccine this season is a reasonably good match to the circulating strains, and also that early epidemic seasons in all previous years of surveillance data were due to the spread of one subtype only.

**The time series of US weekly confirmed cases of influenza between 1997 to present.**
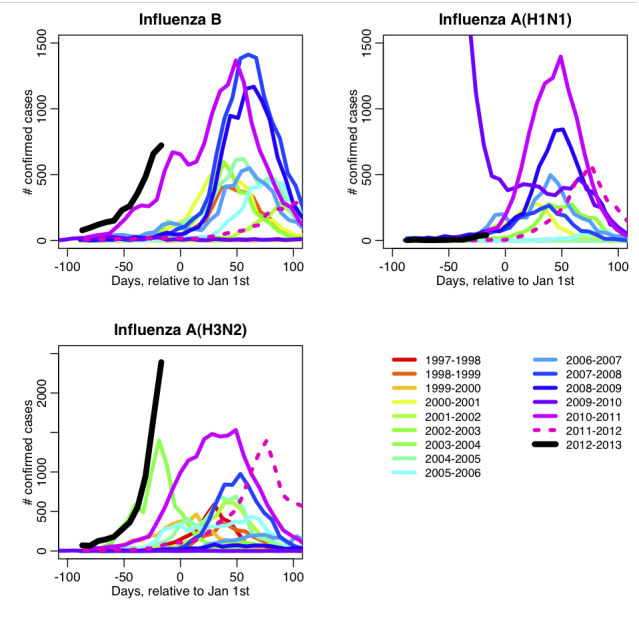
The data for each season is shown relative to Jan 1st of that season. The extraordinarily early rise in cases of influenza A(H3N2) and influenza B this season is made even more unusual by the fact that the simultaneous early epidemic rise of two strains has not hitherto been observed, and the vaccine composition is a good match to the circulating strains.

To calculate the population weighted average climate across US regions, the population and geospatial location information for all urban centers in the US with population greater than 50,000 were obtained from the US Census Bureau (comprising almost 500 population centers, and over 75% of the population of the US) 11. The corresponding weather stations in the vicinity of each population center were identified, and daily climate data from 1997 to 2012 obtained from the National Climate Data Center (NCDC) of the National Oceanic and Atmospheric Administration (NOAA) 12. For each season, we calculated the average temperature over the time period corresponding to the central 90% of the epidemic curve, “T_bef”. For seasons during which the epidemic was suppressed, we calculated T_bef as the average temperature between the start of October to the end of March (the conclusions of this study were not sensitive to the exact date range used to define the epidemic period).

In Figure 2 we show the US population weighted average temperature during winter (January-March) and autumn (September-December) from 1997 to 2012. The winter of early 2012 was unusually warm, whereas temperature in autumn of 2012 was close to the seasonal average of the study period).

**US population weighted average winter and autumn temperatures.**
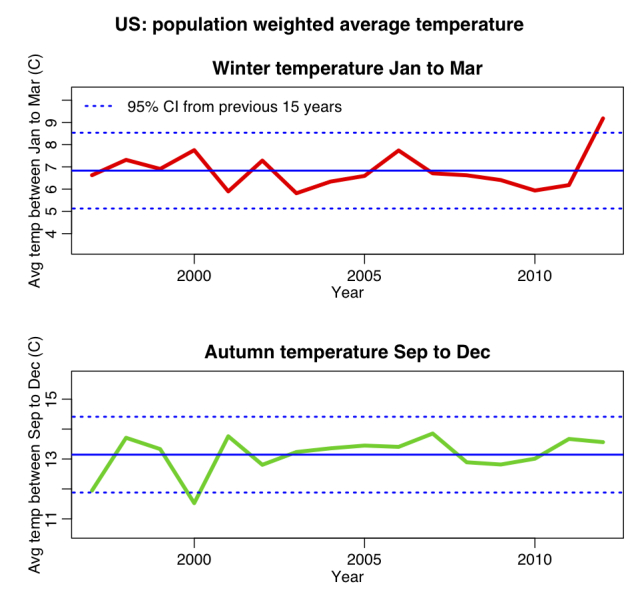
The temperature in early 2012 was significantly higher than average, whereas the temperature during autumn 2012 was consistent with the average.

**Epidemic growth rate as a measure of epidemic severity:** In the early stages of an influenza epidemic, the temporal evolution of incident cases grows exponentially as the effect of increasing incidence on the depletion of the susceptible population remains small 13 14 15. Here we assume that influenza testing rates are relatively constant within each epidemic period, and identify the exponential epidemic phase as the initial portion of the influenza epidemics up to 4 weeks before the time of the peak in incidence^17^ . Maximum likelihood methods are used to fit an exponential curve to that initial epidemic data to determine the initial exponential growth rate, “r” 16. We note that the initial growth rate could not be measured in seasons where the spread of an influenza strain was entirely (or almost entirely) suppressed, and hence we assume in those cases that r=0.

For seasons where the influenza epidemic was not suppressed, we use the NCDC climate data to estimate the average temperature, “T”, over the time period comprising the initial epidemic growth phase.

For each influenza season from 1997-98 to 2012-13, we assessed the rate of exponential rise of the influenza epidemic for all strains, and total ILI mortality during the epidemic. We excluded A(H1N1) data from the 2009-10 pandemic season due to the very unusual dual wave nature of the epidemic curve (although it should be noted that the conclusions of the study remained unchanged even when those data were included).

We found the initial growth rate to be significantly correlated with ILI mortality across seasons (r=45%, p=0.04). In this analysis we thus used the initial growth rate for influenza seasons as an indicator of epidemic severity.

**Partial correlations:** In this analysis we examine correlations between epidemic initial growth rate and other potentially related variables, including temperature during the initial growth period, and the temperature and growth rate of the season before. In assessing the correlations, we control for other potentially confounding variables via the use of partial correlations. For instance, the partial correlation of variables A and B, controlling for variable C, is obtained by first regressing both A and B on C, then assessing the correlation of the residuals from the two regression fits. Multiple variables can be controlled for by regressing both A and B on a linear combination of those variables.

## Results and Discussion

The initial growth rates we estimated for influenza A epidemics are on average 15% higher compared to those we estimated for influenza B epidemics (Student's t-test p=0.03), which is in agreement with results of previous studies 17.

In Figure 3 we show the initial growth rate (r) and epidemic peak timing (t_peak) as a function of the growth rate of the season before (r_bef) and the average temperature the season before (T_bef). Significant associations are seen. In Table 1 we show the partial correlations between the growth rate and time of the peak to the other variables, after adjustment for the accuracy of the vaccine match to the circulating strain (v). We found a significant association between epidemic peak timing and epidemic growth rate when compared to the temperature and growth rate the season before, with very similar patterns seen for both influenza A and influenza B. In particular, mild winters were found to be significantly associated with early and severe epidemics the next season, even after taking into account the epidemic growth rate during that mild winter and the temperature during the next season; we found that when a winter was mild, on average 72% of the time the next epidemic was more severe than average, with epidemic growth rate 40% higher than average, and a peak timing occurring 11 days earlier than average. In addition, the relative likelihood of the following epidemic peaking before January 1^st^ was over 80% higher.

**Dependence of the peak timing and growth rate of influenza epidemics on the growth rate and average temperature of the prior season.**
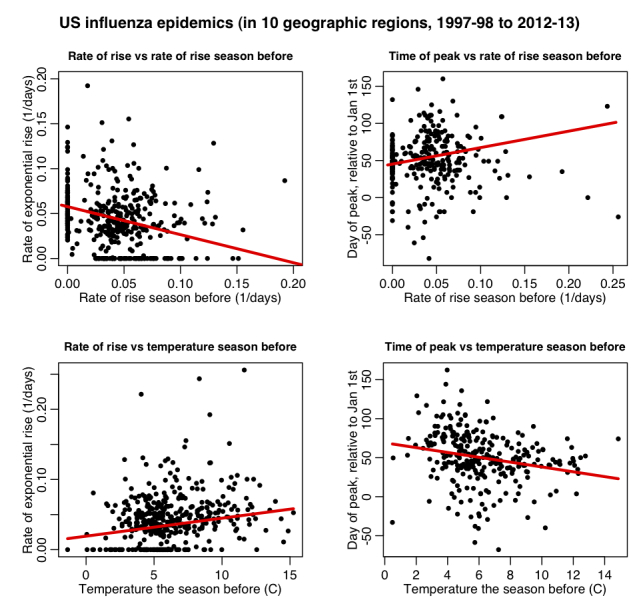

We posit that warm winters are more likely to be succeeded by early and severe influenza seasons due to fewer people being infected due to the warm weather, thereby leaving an unnaturally large fraction of susceptible individuals in the population going into the next season. The severity of the next season could potentially be exacerbated by the early onset, if the onset occurs before most of the population has had the opportunity to be vaccinated.

Our observation that climate patterns can have a profound impact on influenza epidemics beyond just the time frame of the current season will likely open up interesting avenues of further research.

**Table 1:** Correlation of the exponential growth rate (r) and epidemic peak timing (t_peak), to the growth rate and average temperature during the season before, (r_bef) and (T_bef), respectively. The calculation of the correlations takes into account the temperature during the initial epidemic period (T) and the accuracy of the vaccine match (v) for each season under consideration. The numbers in square brackets indicate the 95% CI. The correlations in the influenza A column are the aggregated results of influenza A(H3N2) and A(H1N1) epidemics.

Spearman partial correlations	Influenza A N=280	Influenza B N=150	All Influenza N=430
ρ(r,T|r_bef,T_bef,v)	-0.28[-0.38,-0.18]	-0.39[-0.50,-0.26]	-0.35[-0.43,-0.28]
ρ(r,r_bef|T,T_bef,v)	-0.06[-0.16,0.05]	-0.11[-0.24,0.03]	-0.06[-0.15,0.02]
ρ(t_peak,r_bef|T,T_bef,v)	-0.07[-0.19,0.06]	0.09[-0.06,0.24]	-0.06[-0.16,0.04]
ρ(r,T_bef|T,r_bef,v)	0.15[0.04,0.25]	0.28[0.15,0.40]	0.17[0.09,0.25]
ρ(t_peak,T_bef|T,r_bef,v)	-0.18[-0.30,-0.05]	-0.31[-0.44,-0.17]	-0.11[-0.20,-0.01]
